# Effect of Pore Shape on Mechanical Properties of Porous Shape Memory Alloy

**DOI:** 10.3390/mi13040566

**Published:** 2022-03-31

**Authors:** Bingfei Liu, Yaxuan Pan

**Affiliations:** 1 Science and Technology Innovation Research Institute, Civil Aviation University of China, Tianjin 300300, China; 2 Aviation Engineering Institute, Civil Aviation University of China, Tianjin 300300, China; yaxuanpan2022@126.com

**Keywords:** shape memory alloy, aspect ratio, porosity, constitutive model

## Abstract

Porous shape memory alloys (SMAs) have been widely used in the aerospace, military, medical, and health fields due to its unique mechanical properties such as superelasticity, biocompatibility, and shape memory effect. In this work, the pore shape was considered in the constitutive model of the porous SMAs by respectively introducing the parameter of aspect ratio and for different pore shapes including oblate, sphere, and prolate shapes, so the expression of Young’s modulus for the porous SMA can be derived. Then, the constitutive model for such a porous shape memory alloy was established. When the porosity was zero, the model can be degenerated to the dense case. The stress–strain curves for the porous SMA with a porosity of 13% with different aspect ratio are then given. Numerical results showed good agreement with the published experimental data that proved the validation of the model. Based on the proven constitutive model, the properties of porous SMA with different porosity and pore shapes are discussed. The results showed that the pore shapes and the porosities had a big effect on the stress–strain curves for the porous shape memory, while with the increasing porosities, the Young’s modulus and the hysteresis both decreased. With the same porosities, the Young’s modulus and hysteresis loop of SMA with round pores were the largest, while the Young’s modulus and hysteresis loop were the smallest when r=0.1, and they were greater when r=0.75 than when r=10. It can be seen that the closer to the circle, the better the performance of the material.

## 1. Introduction

Porous shape memory alloys have been widely used in various fields including the aerospace, military, medical, and health fields [1,2,3,4] due to their unique mechanical properties including superelasticity, shape memory effect, biocompatibility, low density, high porosity, and high permeability [5,6,7].

The mechanical properties of porous SMA have been given much attention [8]. Regarding the experiments and simulation work on the porous SMA, the influence of pores on the mechanical properties such as Young’s modulus and strength of the porous SMA has been studied in recent years [9]. Xu et al., prepared SMA with different porosities by the microwave sintering method and studied the effects of porosity on microstructure, hardness, compressive strength, bending strength, elastic modulus, phase transition temperature, and superelasticity of porous SMA [10]. The results showed that with the increase in porosity, the compressive strength, elastic modulus, bending strength, and superelasticity of porous SMA decreased. Zhao et al., manufactured SMA with constant porosity and pore diameter and found that its damping performance could be adjusted with the change in porosity and pore diameters [11]. Zhang et al., successfully prepared porous SMA by combining one-step spark plasma sintering technology with space retainer technology [12]. It was found that the superelastic recovery strain ratio of porous SMA could be increased to more than 90%, but with the further increase in training periods, the curve presents an obvious stepped stress platform, indicating the collapse of pores. In addition, with the increase in porosities and pore sizes, the elastic modulus and compressive strength of porous SMA decrease. Gur et al., compared the simulation results of nano-porous NiTi with different pore structures with those of dense NiTi alloys. The simulation results showed that the martensite phase fraction and phase transition temperature increased significantly with the increase in porosities; moreover, the stress–strain response changed significantly, and residual strain and hysteretic energy dissipation capacity increased significantly with the increase in porosity [13]. Kim et al. studied the effect of porosity on the mechanical properties of the porous stent by the compression test [14]. Galimzyanov et al., used non-equilibrium molecular dynamic simulation methods to study the influence of the degree of porosity of porous amorphous titanium nickel ester on mechanical properties under uniaxial tension, uniaxial compression, and uniform shear loads [15].

Regarding the theoretical work of the porous SMA, Zhao et al. established a macroscopic compression behavior model of porous SMA and predicted the effective elastic and superelastic behavior of porous shape memory alloys based on the assumption of the stress–strain curve by using the Eshelby heterogeneous inclusion method [16]. In addition, they studied two types of pore connectivity and compared the stress–strain curves of porous shape memory alloys with spherical pores and elliptical pores [17]. Based on the micromechanical method and thermodynamic theory, a constitutive model of macroscopic mechanical behavior of porous shape memory alloys was established [18]. According to the phase transition function of dense SMA, considering the hydrostatic stress of porous SMA, the phase transition function of porous SMA is given. Olsen et al., proposed a new constitutive model of porous SMA based on the Gurson–Tvergaard–Needleman formula. The main results showed that the stress of phase transformation and plastic yield will decrease, even if the pores are relatively small [19]. In addition, the existence of micropores leads to a reduction in the area of the stress–strain hysteresis curve, thus affecting the energy dissipation in hyperelastic cycle. Considering Gibbs free energy, Xu et al. established a phenomenological constitutive model that can be used to predict the mechanical behavior of FGP-SMA by using thermodynamic theory and a new transformation function considering the influence of hydrostatic stress was proposed [3]. Zheng et al., studied the effect of a surface modified porous titanium implant and different porosity and aperture [20].

However, most of the above studies are spherical porous SMA. There is no detailed theory about the effect of pore shape on the mechanical behavior of porous SMA, especially in the case of different pore ratios. Although the above practical and theoretical research work on porous SMA have been carried out, at present, no scholars have considered the influence of pore shape on the properties of porous SMA, and the constitutive relationship considering pore shape has not been established. Qiu found that the influence of pore shape on metal materials is very important and demonstrated the great influence of pore shape on metal materials [21]. Therefore, it is necessary to study the influence of pore shape on the properties of SMA materials. In light of this, under the assumption that all pore shapes in the whole material are consistent and evenly distributed in the material, not only is the influence of porosity on modulus of porous SMA discussed, but also the influence of pore shape on the modulus and the stress–strain relationship of porous SMA is established in this work.

## 2. Theoretical Model of Porous SMA with Different Pore Shapes

### 2.1. Modulus

**Figure 1:**
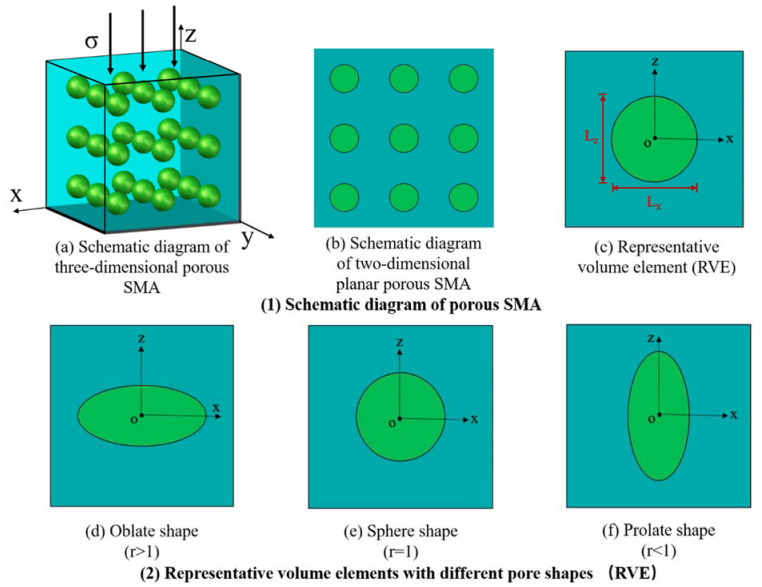
Schematic diagram of the pore shape.

For porous SMA, it is regarded as a uniform porous material with uniform pore shape and uniform orientation, as shown in Figure 1a, where the pores are evenly distributed in space, as shown in Figure 1b. In light of this, when analyzing its mechanical properties, the representative volume unit (RVE), as shown in Figure 1c, can be selected, where the center of the circle is defined as O, the horizontal axis is the x axis, the vertical direction is the z axis, and $L_{x}$, $L_{z}$ represent the diameters of the x axis and z axis of the ellipse, respectively, then $r=L_{x}/L_{z}$, called the aspect ratio, can be defined to describe the change in pores, from the Figure 1d oblate shape ($L_{x}>L_{z}$) to the Figure 1e sphere shape ($L_{x}=L_{z}$) to the Figure 1f prolate shape ($L_{x}<L_{z}$).

The porous SMA is assumed to be the composite of the SMA matrix and pore inclusions, which can be shown in Figure 1. It is assumed that the matrix and inclusion are linearly isotropic, and the elastic modulus is $C_{ijkl}^{0}$ and $C_{ijkl}^{1}$, respectively. The subscript 1 indicates the pore inclusion phase, and the subscript 0 indicates the SMA matrix phase. In order to facilitate the analysis, the material composed only of the matrix with the elastic modulus $C_{ijkl}^{0}$ was introduced as the reference. Therefore, the equivalent applied stress $\sigma_{ij}$ and the strain of composite $\varepsilon_{ij}$ and reference material $\varepsilon_{ij}^{0}$ are expressed by

(1) $$\begin{array}{l} \sigma_{ij}=C_{ijkl}\varepsilon_{kl}\\ \sigma_{ij}=C_{ijkl}^{0}\varepsilon_{kl}^{0} \end{array}$$

where $\sigma_{ij}$ and $\varepsilon_{ij}^{0}$ represent the average stress and strain of the matrix, respectively.

When the inclusion with volume fraction $f_{v}$ is inserted into the reference material of the pure matrix, the average stress of matrix will have a disturbance of ${\tilde{\sigma }}_{ij}$, resulting in the strain disturbance of ${\tilde{\varepsilon }}_{ij}$ on the basis of $\varepsilon_{ij}^{0}$, which can be expressed by

(2) $$\sigma_{ij}+{\tilde{\sigma }}_{ij}=C_{ijkl}^{0}(\varepsilon_{kl}^{0}+{\tilde{\varepsilon }}_{kl})$$

For the average stress of pore inclusions, because the specific orientation of pores will produce a certain amount of stress–strain disturbance $\sigma_{ij}^{pt}$ and $\varepsilon_{ij}^{pt}$, it can be obtained by Eshelby’s equivalence principle

(3) $$\begin{array}{ll} \sigma_{ij}^{1} & =\sigma_{ij}+{\tilde{\sigma }}_{ij}+\sigma_{ij}^{pt}\\ & =C_{ijkl}^{1}(\varepsilon_{kl}^{0}+{\tilde{\varepsilon }}_{kl}+\varepsilon_{kl}^{pt})\\ & =C_{ijkl}^{0}(\varepsilon_{kl}^{0}+{\tilde{\varepsilon }}_{kl}+\varepsilon_{kl}^{pt}-\varepsilon_{kl}^{*}) \end{array}$$

where $\varepsilon_{kl}^{*}$ is the equivalent transformation strain of the pore inclusion.

From Equations (2) and (3), $\sigma_{ij}^{pt}$ can be expressed as

(4) $$\sigma_{ij}^{pt}=C_{ijkl}^{0}(\varepsilon_{kl}^{pt}-\varepsilon_{kl}^{*})$$

The stress of thee matrix and pore inclusion shall be balanced with the external stress $\sigma_{ij}$, that is

(5) $${\tilde{\sigma }}_{ij}=-f_{v}{\tilde{\sigma }}_{ij}^{pt}$$

In addition, ${\tilde{\sigma }}_{ij}$ also satisfies the following equation

(6) $${\tilde{\sigma }}_{ij}=C_{ijkl}^{0}{\tilde{\varepsilon }}_{kl}$$

where ${\tilde{\varepsilon }}_{ij}$ is given by

(7) $${\tilde{\varepsilon }}_{ij}=-f_{v}(\varepsilon_{ij}^{pt}-\varepsilon_{ij}^{*})$$

which has also been derived by Takao et al. [22]. Then, it could be obtained that

(8) $$\varepsilon_{ij}=\varepsilon_{ij}^{0}+{\tilde{\varepsilon }}_{ij}+f_{v}\varepsilon_{ij}^{pt}=\varepsilon_{ij}^{0}+f_{v}\varepsilon_{ij}^{*}$$

Which is substituted into Equation (1) to obtain the following relation

(9) $$C_{ijkl}(\varepsilon_{kl}^{0}+f_{v}\varepsilon_{kl}^{*})=C_{ijkl}^{0}\varepsilon_{kl}^{0}$$

Since the matrix and inclusion are isotropic, their elastic modulus can be expressed by the bulk modulus, shear modulus, and Kronecker delta $\delta_{ij}$, as follows

(10) $$\begin{array}{l} C_{ijkl}^{0}=\kappa_{0}\delta_{ij}\delta_{kl}+\mu_{0}(\delta_{ik}\delta_{jl}+\delta_{il}\delta_{jk}-2\delta_{ij}\delta_{kl}/3)\\ C_{ijkl}^{1}=\kappa_{1}\delta_{ij}\delta_{kl}+\mu_{1}(\delta_{ik}\delta_{jl}+\delta_{il}\delta_{jk}-2\delta_{ij}\delta_{kl}/3) \end{array}$$

Then, combine Equation (7) to obtain

(11) $$\begin{array}{l} \varepsilon_{11}^{*}=\left[a_{1}(\varepsilon_{11}^{0}+{\tilde{\varepsilon }}_{11})-a_{2}(\varepsilon_{22}^{0}+{\tilde{\varepsilon }}_{22}+\varepsilon_{33}^{0}+{\tilde{\varepsilon }}_{33})\right]/a\\ \varepsilon_{22}^{*}=\left[2a_{3}(\varepsilon_{11}^{0}+{\tilde{\varepsilon }}_{11})+(a_{4}+a_{5}a)(\varepsilon_{22}^{0}+{\tilde{\varepsilon }}_{22})+(a_{4}-a_{5}a)(\varepsilon_{33}^{0}+{\tilde{\varepsilon }}_{33})\right]/2a\\ \varepsilon_{22}^{*}=\left[2a_{3}(\varepsilon_{11}^{0}+{\tilde{\varepsilon }}_{11})+(a_{4}-a_{5}a)(\varepsilon_{22}^{0}+{\tilde{\varepsilon }}_{22})+(a_{4}+a_{5}a)(\varepsilon_{33}^{0}+{\tilde{\varepsilon }}_{33})\right]/2a\\ \varepsilon_{12}^{*}=-\frac{\varepsilon_{12}^{0}+{\tilde{\varepsilon }}_{12}}{2S_{1212}+\mu_{0}/(\mu_{1}-\mu_{0})}\\ \varepsilon_{23}^{*}=-\frac{\varepsilon_{23}^{0}+{\tilde{\varepsilon }}_{23}}{2S_{2323}+\mu_{0}/(\mu_{1}-\mu_{0})}\\ \varepsilon_{13}^{*}=-\frac{\varepsilon_{13}^{0}+{\tilde{\varepsilon }}_{13}}{2S_{1313}+\mu_{0}/(\mu_{1}-\mu_{0})} \end{array}$$

where components of Eshelby’s $S_{ijkl}$ are

(12) $$\begin{array}{l} S_{1111}=\frac{1}{2(1-\nu_{0})}\left\{1-2\nu_{0}+\frac{3r^{2}-1}{r^{2}-1}-\left[1-2\nu_{0}+\frac{3r^{2}}{r^{2}-1}\right]g\right\}\\ S_{2222}=S_{3333}=\frac{3}{8(1-\nu_{0})}\frac{r^{2}}{r^{2}-1}+\frac{g}{4(1-\nu_{0})}\left[1-2\nu_{0}-\frac{9}{4(r^{2}-1)}\right]\\ S_{2233}=S_{3322}=\frac{1}{4(1-\nu_{0})}\left\{\frac{r^{2}}{2r^{2}-1}-\left[1-2\nu_{0}+\frac{3}{4(r^{2}-1)}\right]g\right\}\\ S_{2211}=S_{3311}=-\frac{1}{2(1-\nu_{0})}\frac{r^{2}}{r^{2}-1}+\frac{g}{4(1-\nu_{0})}\left\{\frac{3r^{2}}{r^{2}-1}-(1-2\nu_{0})\right\}\\ S_{1122}=S_{1133}=\frac{1}{2(1-\nu_{0})}\left\{g\left[1-2\nu_{0}+\frac{3}{2(r^{2}-1)})\right]-\left[1-2\nu_{0}+\frac{1}{r^{2}-1}\right]\right\}\\ S_{2323}=S_{3232}=\frac{1}{4(1-\nu_{0})}\left\{\frac{r^{2}}{2(r^{2}-1)}+g\left[1-2\nu_{0}-\frac{3}{4(r^{2}-1)}\right]\right\}\\ S_{1212}=S_{1313}=\frac{1}{4(1-\nu_{0})}\left\{1-2\nu_{0}-\frac{r^{2}+1}{r^{2}-1}-\frac{g}{2}\left[1-2\nu_{0}-\frac{3(r^{2}+1)}{r^{2}-1}\right]\right\} \end{array}$$

where $v_{0}$ is the Poisson’s ratio of the SMA matrix, and g corresponding to r is given by

(13) $$g=\{\begin{array}{cc} \frac{r}{{\left(r^{2}-1\right)}^{\frac{3}{2}}}\left[r{\left(r^{2}-1\right)}^{\frac{1}{2}}-\mathrm{arccosh}r\right] & r<1\\ \frac{2}{3} & r=1\\ \frac{r}{{\left(1-r^{2}\right)}^{\frac{3}{2}}}\left[\arccos r-r{\left(1-r^{2}\right)}^{\frac{1}{2}}\right] & r>1 \end{array}$$

The constants $a,a_{1},a_{2,}a_{3},a_{4},a_{5}$, depending on $\kappa_{1},\kappa_{0},\mu_{1},\mu_{0}$, which are the bulk modulus and shear modulus of pore inclusion and matrix, respectively,

(14) $$\begin{array}{l} a_{1}=6(\kappa_{1}-\kappa_{0})(\mu_{1}-\mu_{0})(S_{2222}+S_{2233}-1)-2(\kappa_{0}\mu_{1}-\kappa_{1}\mu_{0})+6\kappa_{1}(\mu_{1}-\mu_{0})\\ a_{2}=6(\kappa_{1}-\kappa_{0})(\mu_{1}-\mu_{0})S_{1133}+2(\kappa_{0}\mu_{1}-\kappa_{1}\mu_{0})\\ a_{3}=-6(\kappa_{1}-\kappa_{0})(\mu_{1}-\mu_{0})S_{3311}-2(\kappa_{0}\mu_{1}-\kappa_{1}\mu_{0})\\ a_{4}=6(\kappa_{1}-\kappa_{0})(\mu_{1}-\mu_{0})(S_{1111}-1)+2(\kappa_{0}\mu_{1}-\kappa_{1}\mu_{0})+6\mu_{1}(\kappa_{1}-\kappa_{0})\\ a_{5}=1/\left[S_{3322}-S_{3333}+1-\mu_{1}/(\mu_{1}-\mu_{0})\right]\\ a=6(\kappa_{1}-\kappa_{0})(\mu_{1}-\mu_{0})\left[2S_{1133}S_{3311}-(S_{1111}-1)(S_{3322}+S_{3333}-1)\right]\\ \;+2(\kappa_{0}\mu_{1}-\kappa_{1}\mu_{0})\left[2(S_{1133}+S_{3311})+(S_{1111}-S_{3322}-S_{3333})\right]\\ \;-6(\kappa_{1}-\kappa_{0})(\mu_{1}-\mu_{0})(S_{1111}-1)-6\mu_{1}(\kappa_{1}-\kappa_{0})(S_{2222}+S_{2233}-1)-6\kappa_{1}\mu_{1} \end{array}$$

When inclusions are uniformly distributed in three-dimensional space, it is assumed that the composite, as a whole, is macroscopically isotropic. The stress or strain can be decomposed into hydrostatic and deviatoric parts, resulting in the effective bulk modulus and shear modulus, respectively.

From Equation (7), the hydrostatic and deviatoric parts can be found by

(15) $$\begin{array}{l} {\tilde{\varepsilon }}_{kk}=(1/p_{1}-1)\varepsilon_{kk}^{0}\\ {\tilde{\varepsilon }}_{12}=(1/q_{1}-1)\varepsilon_{12}^{0} \end{array}$$

Then

(16) $$\begin{array}{l} \varepsilon_{kk}^{*}=p\varepsilon_{kk}^{0}\\ \varepsilon_{12}^{*}=q\varepsilon_{12}^{0} \end{array}$$

where

(17) $$\begin{array}{l} p=p_{2}/p_{1}\\ q=q_{2}/q_{1}\\ p_{1}=1+f_{v}\left[\begin{array}{l} 2(S_{1122}+S_{2222}+S_{2233}-1)(a_{3}+a_{4})\\ +(S_{1111}+2S_{2211}-1)(a_{1}-2a_{2}) \end{array}\right]/3a\\ p_{2}=\left[a_{1}-2(a_{2}-a_{3}-a_{4})\right]/3a\\ q_{1}=1-f_{v}\left\{\begin{array}{l} \frac{2}{5}\frac{2S_{1212}-1}{2S_{1212}+\mu_{0}/(\mu_{1}-\mu_{0})}+\frac{1}{3}\frac{2S_{2323}-1}{2S_{2323}+\mu_{0}/(\mu_{1}-\mu_{0})}\\ -\frac{1}{15a}\left[\begin{array}{l} \left(S_{1122}-S_{2233})(2a_{3}-a_{4}+a_{5}a\right)\\ +2(S_{1111}-S_{2211}-1)(a_{1}+a_{2})\\ +(S_{1122}-S_{2222}+1)(2a_{3}-a_{4}-a_{5}a) \end{array}\right] \end{array}\right\}\\ q_{2}=-\frac{2}{5}\frac{1}{2S_{1212}+\mu_{0}/(\mu_{1}-\mu_{0})}-\frac{1}{3}\frac{1}{2S_{2323}+\mu_{0}/(\mu_{1}-\mu_{0})}\\ \;+\frac{1}{15a\left[2(a_{1}+a_{2}-a_{3})+a_{4}+a_{5}a\right]} \end{array}$$

Combined with Equation (12), the average disturbance stress in the matrix is obtained by

(18) $$\begin{array}{l} {\tilde{\sigma }}_{kk}=(1/p_{1}-1)\sigma_{kk}\\ {\tilde{\sigma }}_{12}=(1/q_{1}-1)\sigma_{12} \end{array}$$

Then, the effective bulk modulus κ and shear modulus μ of the porous composite can be obtained from Equation (9)

(19) $$\begin{array}{l} \frac{\kappa }{\kappa_{0}}=\frac{1}{1+f_{v}p}\\ \frac{\mu }{\mu_{0}}=\frac{1}{1+f_{v}q} \end{array}$$

$\kappa_{0}$ and $\mu_{0}$ of the SMA matrix can be determined, respectively, by

(20) $$\kappa_{0}=\frac{E_{SMA}^{0}}{3(1-2\nu_{0})}$$

(21) $$\mu_{0}=\frac{E_{SMA}^{0}}{2(1+\nu_{0})}$$

where $v_{0}$ is the Poisson’s ratio of the SMA matrix and $E_{SMA}^{0}$ is the Young’s modulus of the SMA matrix. For the porous SMA with different specified pore shape, its Young’s modulus can be obtained from the following expression,

(22) $$E_{SMA}^{p}=\frac{9\kappa \mu }{3\kappa +\mu }$$

### 2.2. Constitutive Model

For the uniaxial loading case, the one-dimensional unified constitutive model degraded from three-dimensional model of Lagoudas is applied here [23],

(23) $$\dot{\varepsilon }={\dot{\sigma }}_{ij}:{Μ}^{p}+\Lambda_{ij}\dot{\xi }+\alpha^{p}\Delta \dot{T}$$

(24) $$\Lambda_{ij}=\left\{ \begin{array}{ll} \frac{3}{2}H^{p}{\left(\sigma_{ij}\right)}^{-1}\sigma^{\prime } & ,\dot{\xi }>0\\ H^{p}{\left(\varepsilon^{t}\right)}^{-1}\varepsilon_{ij}^{t} & ,\dot{\xi }<0 \end{array}\right.$$

where $H^{p}$ is the maximum phase transformation strain and $M^{p}={\left(E_{SMA}^{p}\right)}^{-1}$ is the elastic flexibility tensor; and $E_{SMA}^{p}$ is the Young’s modulus of porous SMA, which is expressed as follows

(25) $$E_{SMA}^{p}=E_{A}^{p}+\xi^{p}(E_{M}^{p}-E_{A}^{p})$$

where $\xi^{p}$ is the martensitic volume fraction of porous SMA, which is related to the critical stresses of porous SMA.

(26) $$\xi^{p}=\{\begin{array}{cc} 0 & \sigma_{e}\leq \sigma_{s}^{p}\\ \frac{\sigma_{e}-\sigma_{s}^{p}}{\sigma_{f}^{p}-\sigma_{s}^{p}} & \sigma_{s}^{p}\leq \sigma_{e}\leq \sigma_{f}^{p}\\ 1 & \sigma_{f}^{p}\leq \sigma_{e} \end{array}$$

where $\sigma_{e}$ is the effective stress, which is expressed as follows:

(27) $$\sigma_{e}=\sqrt{3J_{2}}$$

where $J_{2}$ represents the invariant of the second deviation of stress, which is expressed by

(28) $$J_{2}=\frac{1}{2}\sigma^{\prime }:\sigma^{\prime }$$

(29) $$\sigma^{\prime }=\sigma_{ij}-\frac{1}{3}\sigma_{kk}$$

For the uniaxial loading,

(30) $$\sigma_{ij}=\left(\begin{array}{ccc} \sigma_{11} & 0 & 0\\ 0 & 0 & 0\\ 0 & 0 & 0 \end{array}\right)$$

$\alpha^{p}$ is the thermal expansion coefficient, which can be expressed as follows:

$$\alpha^{p}=\alpha_{A}^{p}+\xi^{p}(\alpha_{M}^{p}-\alpha_{A}^{p})$$

where $\alpha_{M}^{p}$ and $\alpha_{A}^{p}$ represent the thermal expansion coefficients of martensite and austenite, respectively. $\Delta \dot{T}$ is the increment of temperature difference, $\sigma_{s}^{p}$, $\sigma_{f}^{p}$ (the lower corner marks s and f represent the starting point and the ending point of phase transformation, respectively.)

The critical stress of porous SMA is affected by porosity, $f_{v}$, can be assumed as a linear relationship with the critical stress of solid SMA, and $\sigma_{s}^{D}$ or $\sigma_{f}^{D}$, which could be assumed and expressed as follows

(31) $$\sigma_{s}^{p}=(1-2f_{v})\sigma_{s}^{D}$$

(32) $$\sigma_{f}^{p}=(1-2f_{v})\sigma_{f}^{D}$$

## 3. Numerical Analysis Results

### 3.1. Young’s Modulus of SMA with Different Pore Shapes and Porosities

In order to analyze the influence of pores including the volume fraction and aspect ratio on the mechanical properties of SMA, the mechanical parameters of solid SMA in the references are quoted here [17], as shown in Table 1. It is assumed that the temperature and the Poisson’s ratio of the material remains constant (i.e., $\nu_{M}=\nu_{A}=\nu =0.3$).

**Figure 2:**
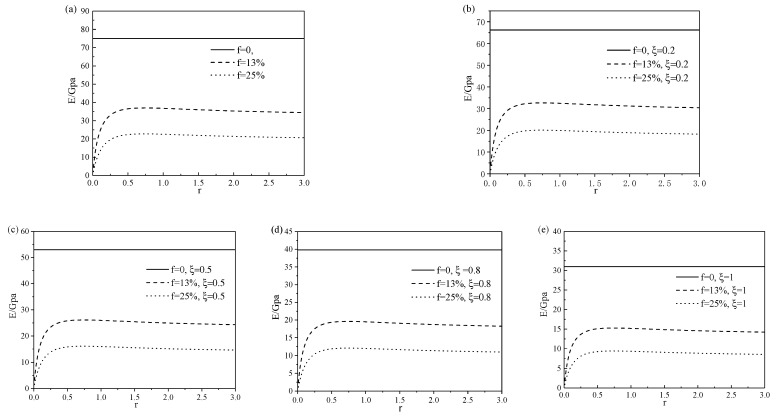
Relationship between Young’s modulus in different phase states and the aspect ratio of porous SMA at porosities of 0%, 13%, and 25%: (**a**) pure austenitic phase, (**b**) mixed martensite and austenite phase at ξ=20%, (**c**) mixed martensite and austenite phase at ξ=50%, (**d**) mixed martensite and austenite phase at ξ=80%, and (**e**) pure martensite phase.

The influence of pore aspect ratio on the Young’s modulus of porous SMA under different porosity is shown in Figure 2. Figure 2a–e show the influence of pore aspect ratio on the Young’s modulus of porous SMA at different phase transformation stages. It can be seen from the figure that when the aspect ratio was between 0 and 0.25, the Young’s modulus changed greatly with the increase in aspect ratio, while when the aspect ratio exceeded 0.25, the aspect ratio had little effect on the Young’s modulus and the modulus was the largest when r=0.75. For the same aspect ratio, the Young’s modulus decreased with the increase in porosity.

**Figure 3:**
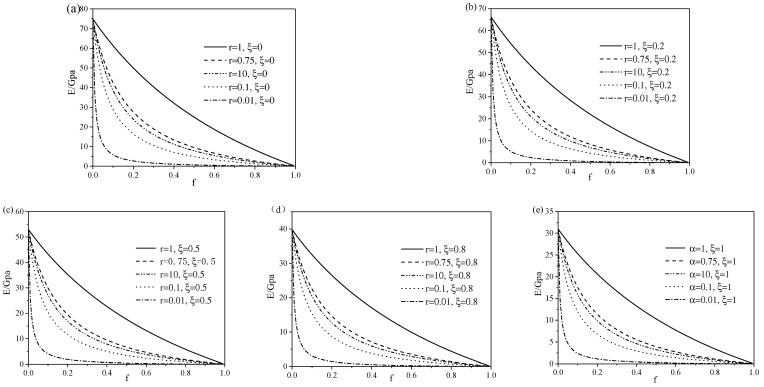
Relationship between Young’s modulus in different phase states and the porosity of porous SMA with the aspect ratio of 0.01, 0.1, 0.75, 1, and 10: (**a**) pure austenitic phase, (**b**) mixed martensite and austenite phase at ξ=20%, (**c**) mixed martensite and austenite phase at ξ=50%, (**d**) mixed martensite and austenite phase at ξ=80%, and (**e**) pure martensite phase.

Then, similar to Figure 2, when the aspect ratio was constant, the relationship between the Young’s modulus and porosity in different phase states were plotted in Figure 3. For porous SMA in any phase transformation state, the Young’s modulus of porous SMA with round pores (r=1) was always the largest, followed by r=0.75, r=10, r=0.1, and r=0.01. When r=0.1 and r=10, the Young’s modulus was close. It can be seen that the closer to the round hole, the greater the Young’s modulus of the material. In addition, when the porosity was between 0 and 0.1, the Young’s modulus decreased rapidly with the increase in porosity $f_{v}$, while when $f_{v}$ exceeded 0.1, the effect of porosity on Young’s modulus decreased. Furthermore, for the case of r=0.1 and r=10, which actually had the same shape in appearance but were situated in different orientations in uniaxial compression test, it can be observed that the Young’s modulus of porous SMA for the former was smaller than the latter.

**Figure 4:**
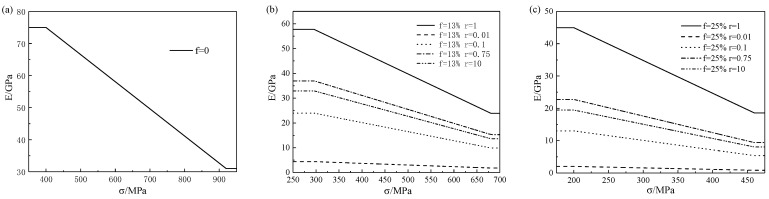
Comparison of the relationship between the Young’s modulus of five types of porous SMA with effective stress at different porosities of (**a**) 0, (**b**) 13%, and (**c**) 25%.

The change in Young’s modulus of porous SMA under different loads is shown in Figure 4. It can be seen from Figure 4 that in the phase transition stage, the Young’s modulus decreased with the increase in load, but remained unchanged in the complete elastic stage. For different pore shapes, the Young’s modulus was the largest when r=1 was the largest, followed by r=0.75, r=0.1, r=10, and the smallest when r=0.01.

### 3.2. Stress–Strain Relationship of SMA with Different Pore Shapes and Porosities

**Figure 5:**
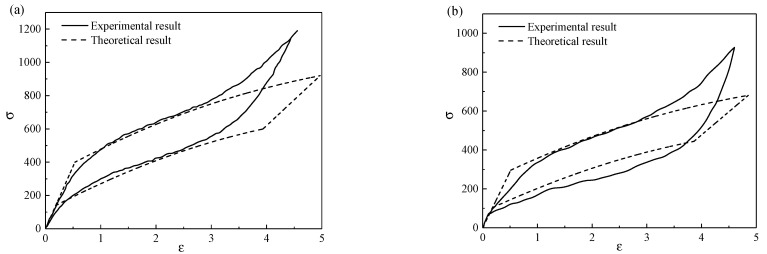
Comparisons of the theoretical and experimental results of (**a**) solid SMA and (**b**) porous SMA with a porosity of 13%.

In order to verify the correctness of the theoretical model in this paper, it is necessary to compare the theoretical results with the experimental results. However, it is difficult to prepare porous SMA with different pore shapes, and there have been few experimental reports on the mechanical behavior of porous SMA with different pore shapes. Due to the lack of experimental data, it is difficult to compare the theoretical curve of stress and strain of porous SMA with the different pore shapes in the experimental results. Therefore, in order to verify the correctness of this model, the constitutive model of porous SMA with spherical pores in this paper was compared with the experimental data of porous SMA with spherical pores [17], as shown in Figure 5. The solid line represents the published experimental result, and the dotted line represents the simulation result in this paper. Obviously, the comparison results of the two cases showed a certain degree of consistency between the theory and the experiment, which verifies the correctness of the model in this paper. In contrast, the warping angle at the top of the hysteretic curve depends on the selected theoretical model.

**Figure 6:**
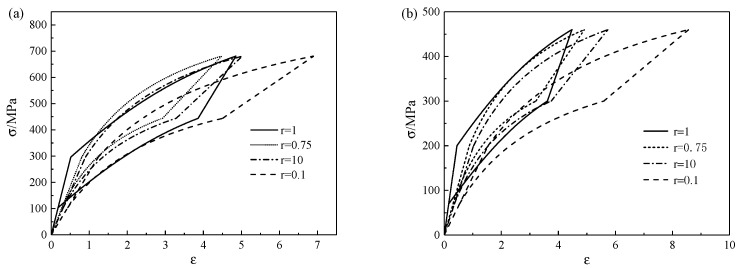
Stress–strain curves of different aspect ratios with porosities of (**a**) 13% and (**b**) 25%.

Next, the influence of various pore shapes on the mechanical properties of porous SMA is further discussed. The stress–strain curves can be obtained when the porosity is 13% and 25%, respectively, as shown in Figure 6. It can be seen from Figure 6 that the hysteresis curve was the largest when r=1, the smallest when r=0.75, and the maximum strain was the largest when r=0.1, but there was little difference in the other three cases. Thus, the pore shape had a great impact on the stress–strain relationship of the porous SMA.

**Figure 7:**
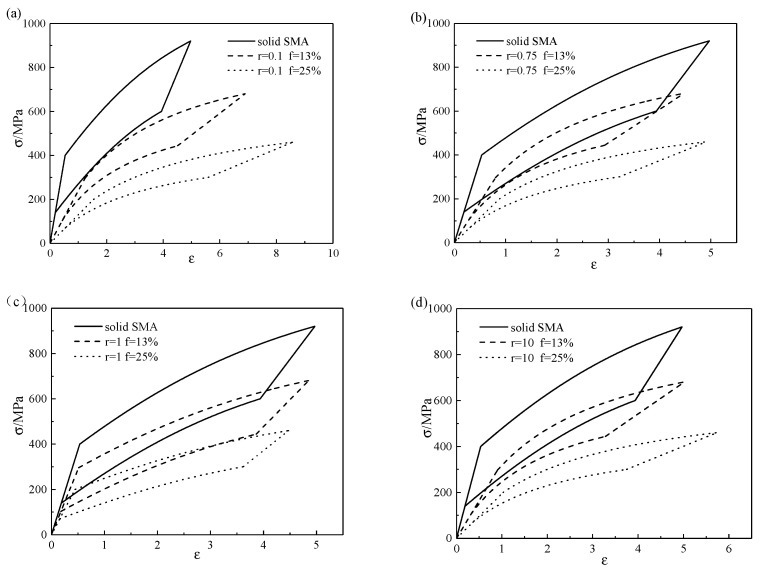
Stress–strain curves of different porosities with aspect ratios of (**a**) r=0.1, (**b**) r=0.75, (**c**) r=1, and (**d**) r=10.

When the pore shape is determined, the stress–strain curves of SMA with different porosities are shown in Figure 7. It can be seen from Figure 7 that with the increase in porosity, the phase transformation stresses of the porous SMA gradually decreased, the hysteretic curve gradually moved down and shrank, and the degree of change was the largest when r=0.1.

## 4. Conclusions

1. The Young’s modulus of porous SMA is related to porosity and pore shape. With the increase in porosity $f_{v}$, the Young’s modulus decreased; when $f_{v}$ was between 0 and 0.1, the influence degree was the largest, and when $f_{v}$ was between 0.1 and 1, it had little impact on the Young’s modulus. When the pore aspect ratio r gradually increased in the range of 0–0.25, the Young’s modulus increased significantly. When r exceeded 0.25 and increased gradually, the Young’s modulus first increased slowly and then decreased slowly, reaching the maximum at about r=0.75.
2. The pore shape and porosity had a great influence on the stress–strain relationship of porous SMA, and the pore shape had an irregular influence on the stress–strain relationship. With the increase in porosity, the critical stresses decreased and the area of the hysteretic curve of the stress–strain relationship decreased.

## Figures and Tables

**Table 1:** Mechanical parameters of solid SMA.

$E_{M}^{s}$	$E_{A}^{s}$	$\sigma_{Ms}^{s}$	$\sigma_{As}^{s}$	$\sigma_{Mf}^{s}$	$\sigma_{Af}^{s}$	H	v
31	75	400	600	920	140	0.02	0.3
